# Integration of proteomics and bioinformatics in traumatic brain injury biomarker discovery

**DOI:** 10.5114/bta/202470

**Published:** 2025-06-30

**Authors:** Mohamed M. Mohamed, El-Sayed A. El-Absawy, Hala M. Ahmed, Mohamed E. Hasan

**Affiliations:** 1 Department of Basic Sciences, Faculty of Physical Therapy, Alryada University for Science and Technology, Egypt; 2 Bioinformatics Department, Genetic Engineering and Biotechnology Research Institute, University of Sadat City, Egypt; 3 Department of Biomedical Equipment, Faculty of Applied Health Sciences Technology, October 6 University, Egypt; 4 Faculty of Health Sciences Technology, Borg Al Arab Technological University, Alexandria, Egypt

**Keywords:** traumatic brain injury (TBI), GFAP, S-100B, UCH-L1, proteomics, bioinformatics, structural biomarkers, AlphaFold

## Abstract

**Background:**

Traumatic brain injury (TBI) is a significant medical crisis with no FDA-approved therapies to improve functional outcomes. Key biomarkers, such as glial fibrillary acidic protein (GFAP), S-100 calcium-binding protein B (S-100B), and ubiquitin C-terminal hydrolase L1 (UCH-L1), are crucial for understanding TBI pathology.

**Materials and methods:**

This study integrates proteomic and bioinformatic approaches to explore established TBI biomarkers’ structural and functional complexities: GFAP, S-100B, and UCH-L1.

**Results:**

Our comprehensive secondary structure and solvent accessibility assessment, conducted with PredictProtein, confirmed the predominance of alpha-helices in GFAP and S-100B, while UCH-L1 displayed a balanced mix of helices (65.00, 67.39, and 40.81%), beta strands (6.20, 0, and 17.94%), and coils (40.81, 17.94, and 41.26%). AlphaFold and I-TASSER were identified as the best servers for full-length tertiary structure prediction for the three target proteins, based on root-mean-square deviation (RMSD), TM-score, and C-score assessments. Protein motif database scans predicted four, eight, and one protein-binding motifs and two, three, and one post-translational modifications for GFAP, S-100B, and UCH-L1, respectively.

**Conclusions:**

GFAP’s role in axonal transport and synaptic plasticity was emphasized through motifs such as Filament and DUF1664. S-100B’s association with neuroinflammation and oxidative stress post-TBI was supported by the S-100/ICaBP-type calcium-binding domain. UCH-L1’s dualistic impact on TBI was further clarified by the Peptidase_C12 motif. This approach deepens our comprehension of these biomarkers and paves the way for targeted diagnostics in TBI.

## Introduction

Traumatic brain injury (TBI) is a heterogeneous condition resulting from an external force on the head, causing brain damage and impairing cognitive, physical, and emotional functions. TBI is a significant cause of mortality and morbidity worldwide, particularly among young and elderly populations. Symptoms vary depending on the severity and location of the injury and may include headache, dizziness, confusion, memory loss, personality changes, and loss of consciousness (Dadas et al. 2018). TBI can also lead to chronic neurological and cognitive disorders, such as epilepsy, Parkinson’s disease, and Alzheimer’s disease (Smith et al. 2013).

TBI diagnosis is based on clinical assessment and neuroimaging modalities, such as computed tomography and magnetic resonance imaging (Cheema et al. 2024). Treatment strategies involve pharmacological, surgical, rehabilitative, and psychological interventions (Maas et al. 2008). Preventative measures include wearing protective equipment, using seatbelts, and enforcing safety regulations (Langlois et al. 2006).

Biomarkers are biological indicators that can be measured to diagnose, monitor, and predict the outcome of TBI. They provide objective and specific information regarding the extent and nature of brain damage, as well as responses to treatment and recovery (Mondello et al. 2021). Biomarker discovery for TBI relies on various approaches and technologies, such as proteomics, transcriptomics, and metabolomics, which enable the analysis of molecular changes in the brain following injury (Zetterberg and Blennow 2016). Several protein biomarkers have been proposed for TBI, including S-100 calciumbinding protein B (S-100B), neuron-specific enolase, tau, and glial fibrillary acidic protein (GFAP), each reflecting different aspects of brain injury and recovery (Papa et al. 2016).

Ubiquitin is a regulatory protein found in all cells of the body. Ubiquitin C-terminal hydrolase L1 (UCH-L1), a specific isoform of ubiquitin, is primarily located in central neurons and the neuroendocrine system but has also been detected in the testis, ovaries, and kidneys (Zetterberg et al. 2010). GFAP, a member of the intermediate filament family of cytoskeletal proteins, provides structural support to neuroglia. Neuroglia help maintain homeostasis, form myelin, and protect neurons in both the peripheral and central nervous systems. GFAP has also been detected in other cell types outside the central nervous system, including Schwann cells, myoepithelial cells, chondrocytes, fibroblasts, and lymphocytes (Posti et al. 2016).

GFAP and UCH-L1 are frequently used together in m-TBI biomarker analysis to measure the different cell types potentially affected by injury. UCH-L1 is associated with more diffuse brain injuries, whereas GFAP is typically elevated in focal injuries (Papa et al. 2012). The UCH-L1 and GFAP proteins are measured and reported separately, with both results needed to obtain a final brain traumatic indicator (BTI) result. A BTI is reported as “positive” if either or both UCH-L1 and GFAP levels exceed the predetermined cutoff (Mitchell et al. 2020).

S-100B, a calcium-binding protein primarily produced by astrocytes, serves as a biomarker for neural distress and plays a dual role in brain function (Michetti et al. 2018). At low concentrations, it promotes neuronal survival and astrocyte proliferation, whereas at high levels, it induces inflammation and neuronal death (Rothermundt et al. 2003; Sorci et al. 2010). S-100B is involved in various neurological disorders, including acute brain injury, neurodegenerative diseases, and psychiatric conditions (Michetti et al. 2018). Although often considered a brain-specific marker, S-100B is also synthesized in other tissues (Gayger-Dias et al. 2023). The protein’s ability to cross the blood-brain barrier remains debated, with recent research emphasizing the role of the glymphatic system in S-100B clearance (Gayger-Dias et al. 2023). S-100B has diverse functions, including the regulation of protein phosphorylation, energy metabolism, and cell proliferation (Sorci et al. 2010). Its levels in biological fluids are used to monitor disease progression; however, its broad involvement reduces specificity (Michetti et al. 2018).

Proteomics and bioinformatics are particularly useful for identifying and validating protein biomarkers for TBI, as proteins play a crucial role in brain function and pathology (Kobeissy et al. 2008). Protein structure prediction is a fundamental aspect of computational biology and bioinformatics, aiming to determine the threedimensional structure of a protein from its amino acid sequence. This field has seen significant advancements with the integration of conventional computational methods and deep learning techniques.

Traditional approaches to protein structure prediction often involve comparative modeling, in which the structure of an unknown protein is inferred based on its similarity to one or more known protein structures. These methods rely heavily on the availability of homologous protein sequences in databases (Jisna and Jayaraj 2021). In recent years, deep learning has revolutionized protein structure prediction. Techniques such as convolutional neural networks and recurrent neural networks have been employed to extract complex features from protein sequences, leading to more accurate predictions.

Accurate protein structure prediction is crucial for various applications, including drug discovery, antibody design, and understanding protein–protein interactions. As the field continues to evolve, computational methods are expected to become even more integral to biological research and medicine (Jisna and Jayaraj 2021).

This study aims to discover biomarkers for TBI using an integrative approach that combines proteomics and bioinformatics. Additionally, it employs systematic *in silico* prediction and analysis of novel biomarker proteins to interpret the structural and functional correlations between known and newly determined protein structures. These findings could be effectively used in further studies as potential candidates for drug targeting.

## Materials and methods

Our methodology for analyzing traumatic brain injury biomarker proteins included predicting conserved regions, domains, secondary structures, three-dimensional structures, post-translational modification (PTM) sites, signatures, and motifs.

### Conserved regions

Multiple sequence alignments of GFAP (NP_002046.1), S-100B (NP_006263.1), and UCH-L1 (NP_004172.2) were performed using BIOEDIT 7.2 software (Hall et al. 2001) to extract conserved regions through hidden Markov model (HMM) profile-profile algorithms and seeded guide trees. BIOEDIT 7.2 is a user-friendly biological sequence alignment editor that provides basic editing, alignment, manipulation, and analysis functionalities for protein sequences and is comparable to the best alignment techniques.

### Molecular evolutionary and phylogenetic analysis

The evolutionary history was inferred using the Neighbor-Joining approach. To increase the probability of accurately observing amino acid sequences in our data, the maximum likelihood method was used to determine the topology and branch lengths of the phylogenetic tree.

MEGA11 (Tamura et al. 2021) represents a significant advancement in computational molecular evolution. It offers a comprehensive suite of tools for constructing time trees of species, pathogens, and gene families, employing rapid relaxed-clock methods to estimate divergence times and confidence intervals. The software has been enhanced with new features, including a Bayesian method for estimating the neutral evolutionary probabilities of alleles using multispecies sequence alignments and a machine learning approach to test for the autocorrelation of evolutionary rates in phylogenies.

### Domain separation

Domain separation is the first step in predicting a three-dimensional protein structure. The NCBI Conserved Domains Database (CDD) (Lu et al. 2020) is a freely accessible tool for annotating sequences with the positions of conserved protein domain footprints, functional sites, and motifs deduced from these footprints.

ThreaDom has been the top prediction server for protein domains in CASP12, CASP13, CASP14, and CASP15. ThreaDomEx, which integrates ThreaDom and DomEx, provides more precise predictions (Wang et al. 2017). ProDom is a comprehensive database of protein domain families derived from a global comparison of protein sequences (Bru et al. 2005). The NCBI CDD also queries the Conserved Domain Database (Marchler-Bauer et al. 2015).

### Secondary structure prediction

Several servers have been utilized for secondary structure prediction, including PredictProtein (Qiu et al. 2020), a meta-service that provides predictions of structural and functional features of proteins, such as secondary structure, solvent accessibility, transmembrane helices, coiled coils, disulfide bonds, and disorder regions. JPred (Drozdetskiy et al. 2015) employs the Jnet algorithm, one of the most accurate methods for secondary structure prediction. PredictProtein and JPred were used to analyze the exposed and buried regions of GFAP, S-100B, and UCH-L1 proteins.

RaptorX, a deep learning-based method, has achieved state-of-the-art performance in contact prediction in CASP12 and CASP13. Other methods, such as PSIPRED, SOPMA, Porter, YASPIN, and PROTEUS, use different neural network architectures and input features to predict secondary structure elements (alpha helices, beta strands, and coils) with varying accuracy depending on sequence quality and protein size.

### Three-dimensional (3-D) structure prediction

Protein structure prediction, a key area in computational biology, involves homology modeling, fold recognition, and *ab initio* methods. Various servers, including I-TASSER (Zhou et al. 2022), Swiss-Model (Waterhouse et al. 2018), Phyre2 (Kelley et al. 2015), and GalaxyWEB (Ko et al. 2012), have been developed for these techniques.

I-TASSER, a top-performing platform in CASP7– CASP14 assessments, uses iterative simulations for full-length atomic model construction. SWISS-MODEL (Waterhouse et al. 2018), a dedicated service for protein structure homology modeling, provides access to a vast collection of experimentally determined protein structures. The Robetta server (Kim et al. 2004) offers automated methods for protein structure analysis and prediction. DeepMind’s AlphaFold (Jumper et al. 2020), the winner of the CASP13 competition, accurately predicts protein structures from amino acid sequences.

#### Model refinement

Web-based tools such as DeepRefiner (Shuvo et al. 2021), GalaxyRefine (Heo et al. 2013), ModRefiner (Xu and Zhang 2011), and 3Drefine (Bhattacharya et al. 2016) refine protein structures using energy minimization and molecular dynamics techniques. These tools enhance both global and local structural features of initial protein models. The refinement process involves optimizing the hydrogen bonding network and applying composite physics- and knowledge-based force fields for atomic-level energy minimization (Feig and Mirjalili 2015). The refined protein structures can be used for various downstream analyses.

#### Model evaluation

Large-scale model quality assessment (QA) techniques are employed alongside model clustering to rank and select protein structural models. Various metrics, such as GDT-TS, GDT-HA, TM-score, Z-score, MolProbity (MP) score, QMEAN score, projected absolute model quality Z-score, clash score, and root-meansquare deviation (RMSD), are used to evaluate refinement category predictions. These metrics assess model quality aspects, including total fold, interatomic contact distributions, and dihedral angle distributions.

The efficacy of automated protein structure prediction methods for GFAP, S-100B, and UCH-L1 was assessed using servers such as GalaxyRefiner (Heo et al. 2013), ModRefiner (Xu and Zhang 2011), ProQ– Protein Quality Predictor (Benkert et al. 2011), ProSAweb (Wiederstein and Sippl 2007), RAMACHANDRAN PLOT Server (Kleywegt and Jones 1996), QMEAN Server for Model Quality Estimation (Studer et al. 2020), TM-Score (Zhang and Skolnick 2004), and SAVES v6.0 (Hooft et al. 1996), a multiprogram that includes ERRAT (Colovos and Yeates 1993), VERIFY 3D (Lüthy et al. 1992), PROVE (Pontius et al. 1996), PROCHECK (Laskowski et al. 1993), and WHATCHECK (Hooft et al. 1996). Additionally, TM-align (Zhang and Skolnick 2005) was used for structural alignment.

### Functional motifs prediction

Motifs and fingerprints are instrumental in identifying distant sequence relationships and facilitating protein–protein interactions (PPI). The PROSITE web server (De Castro et al. 2006; Sigrist et al. 2012), including its enhanced version ScanProsite, was used to match regular expressions with a query sequence. The SMART (Letunic et al. 2021) web server stores sequence information from multiple sequence alignments and represents it using probabilistic models, such as Position-Specific Scoring Matrices (PSSMs), profiles, or HMMs. Several servers like MotifScan (Shao et al. 2012), MotifFinder, InterPro (Mitchell et al. 2015), and Superfamily (Wilson et al. 2009), and visualization tools like CDvist (Adebali et al. 2015) aid in identifying and interpreting functional motifs within the protein.

### Structural classification

The InterPro database (Mitchell et al. 2015) classifies protein sequences into families and identifies significant domains and conserved regions. InterProScan checks sequences against InterPro’s signatures, which are prediction models defining protein families, domains, or functional sites. Protein structural domains are classified in the SCOP database (Andreeva et al. 2014) based on their structures and amino acid sequences. Databases such as CATH (Sillitoe et al. 2021) and PIR (Wu et al. 2003) predict protein function based on structural features, while Superfamily (Wilson et al. 2009) provides annotation and classification of protein domains and families. CATH (Sillitoe et al. 2021) recognizes domains in protein structures from the wwPDB and groups them into evolutionary superfamilies.

### Pathway and systems biology analysis

To elucidate the functional relationships between GFAP, S-100B, and UCH-L1 in TBI, we conducted a structured bioinformatics analysis using the STRING database (version 11.5) (Szklarczyk et al. 2019). The proteins GFAP (ENSP00000253408), S-100B (ENSP00000291700), and UCH-L1 (ENSP00000284440) were queried using their Ensembl identifiers to construct a PPI network. Interactions were predicted using STRING’s default parameters, including a medium confidence threshold (score ≥ 0.4), and integrated evidence from co-expression, experimental datasets, and text mining. Functional enrichment analysis was performed to identify associations with TBI-related pathways, such as neuroinflammation and ubiquitination, using Gene Ontology (GO), Reactome, and WikiPathways annotations. The network topology and interaction scores were visualized using coordinates provided in the STRING output files, and all raw data were cross-validated for consistency.

## Results and discussion

The UniProt Knowledgebase (UniProtKB) was used to retrieve the amino acid sequences of three biomarker proteins: GFAP (accession number NP_002046.1), UCH-L1 (accession number NP_004172.2), and S-100B (accession number NP_006263.1). These proteins were then subjected to *in silico* prediction and threedimensional structural analysis.

## Prediction of the conserved region of GFAP, S100B and UCHL-1

BioEdit 7.2 software was used to assess essential features and predict conserved regions in UCH-L1, S-100B, and GFAP, identifying 5, 1, and 5 conserved segments, respectively. The analysis highlighted significant similarity and crucial roles for these conserved regions, with minimum segment lengths of sixteen and maximum average entropy values of 0.0331 (Table 1).

**Table 1:** Predicted conserved region of UCH-L1, S-100B and GFAP protein of traumatic brain injury using BioEdit

Proteins^a^	Region^b^	Position^c^	Consensus^d^	Segment length^e^	Average entropy (Hx)^f^
UCH-L1	1	61–92	NFRKKQIEELKGQEVSPKVYFMKQTIGNSCGT	32	0.0095
	2	108–123	FEDGSVLKQFLSETEK	16	0.0331
	3	125–144	SPEDRAKCFEKNEAIQAAHD	20	0.0000
	4	146–186	VAQEGQCRVDDKVNFHFILFNNVDGHLY ELDGRMPFPVNHG	41	0.0074
	5	196–223	DAAKVCREFTEREQGEVRFSAVALCKAA	28	0.0000
S-100B	1	22–38	EGDKHKLKKSELKELIN	17	0.0199
GFAP	1	66–130	GFKETRASERAEMMELNDRFASYIEKVRF LEQQNKALAAELNQLRAKEPTKLADVYQAELRELRL	65	0.0094
	2	157–175	RQKLQDETNLRLEAENNLA	19	0.0338
	3	177–194	YRQEADEATLARLDLERK	18	0.0063
	4	251–276	ASSNMHEAEEWYRSKFADLTDAAARN	26	0.0234

a Proteins: The analyzed protein names.

b Region: The conserved sequence region identified within each protein.

c Position: The specific amino acid range where the conserved region is located.

d Consensus: The consensus sequence of the conserved region is based on multiple sequence alignments.

e Segment length: The number of amino acids in the conserved region.

f Average entropy (Hx): A measure of sequence variability within the conserved region, where lower entropy values indicate higher conservation

## Molecular evolutionary and phylogenetic analysis

**Figure 1:**
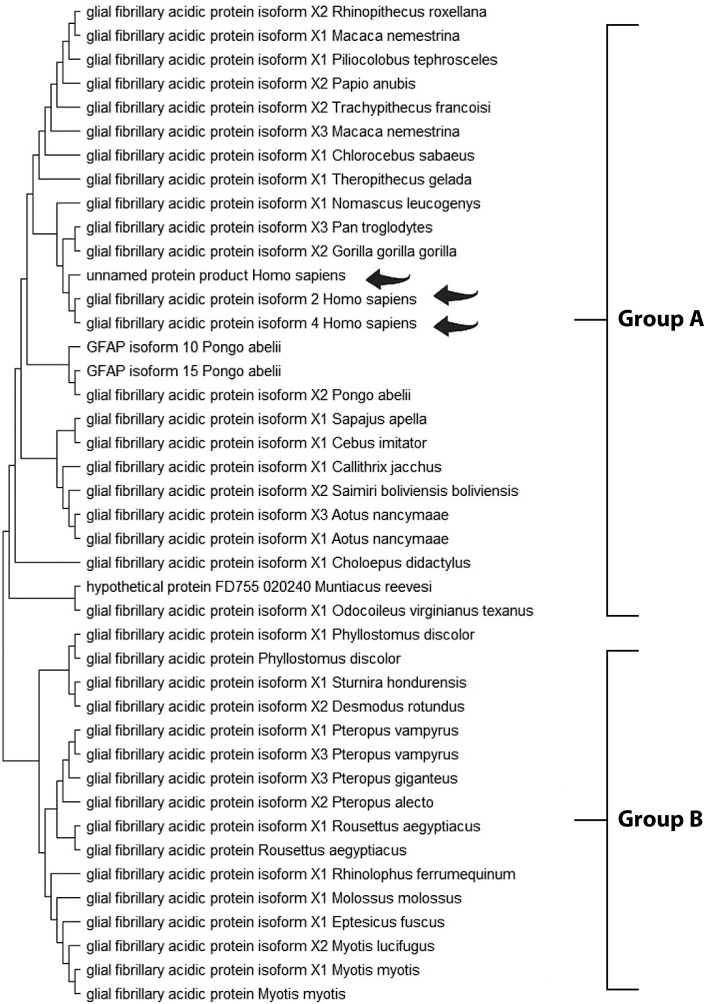
Molecular phylogenetic analysis of the GFAP protein using the maximum likelihood method. The evolutionary history was inferred using the maximum likelihood method and the JTT matrix-based model (Jones et al. 1992). The tree with the highest log likelihood (–2493.15) is shown. Initial trees for the heuristic search were obtained automatically by applying the Neighbor-Joining and BioNJ algorithms to a matrix of pairwise distances estimated using the JTT model, followed by selecting the topology with the highest log likelihood value. The final dataset included 436 positions

**Figure 2:**
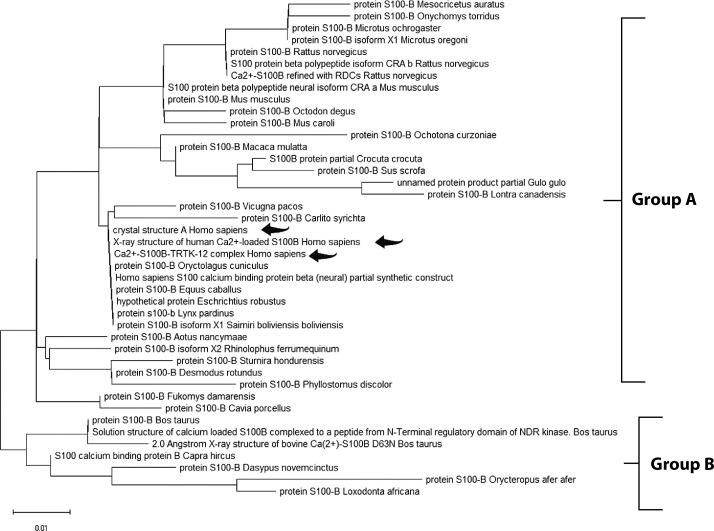
Molecular phylogenetic analysis of the S-100B protein using the maximum likelihood method. The evolutionary history was inferred using the maximum likelihood method and the JTT matrix-based model (Jones et al. 1992). The tree with the highest log likelihood (–517.32) is shown. Initial trees for the heuristic search were obtained automatically by applying the Neighbor-Joining and BioNJ algorithms to a matrix of pairwise distances estimated using the JTT model, followed by selecting the topology with the highest log likelihood value. The final dataset included 92 positions

**Figure 3:**
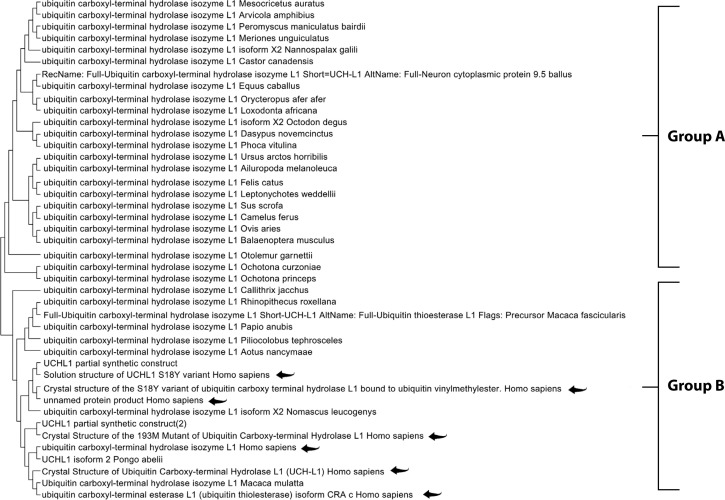
Molecular phylogenetic analysis of the UCH-L1 protein using the maximum likelihood method. The evolutionary history was inferred using the maximum likelihood method and the JTT matrix-based model (Jones et al. 1992). The tree with the highest log likelihood (–1186.09) is shown. Initial trees for the heuristic search were obtained automatically by applying the Neighbor-Joining and BioNJ algorithms to a matrix of pairwise distances estimated using the JTT model, followed by selecting the topology with the highest log likelihood value. The final dataset included 223 positions

The Maximum Likelihood approach, based on the JTT matrix-based model, along with the Neighbor-Joining and UPGMA methods, was used to infer evolutionary history (Tables 2, 3, and 4). For GFAP, two primary groups were identified. Group A comprised primates, including *Homo sapiens, Pan troglodytes,* and *Gorilla gorilla*, demonstrating strong evolutionary conservation. Group B consisted of species from diverse orders, suggesting broader functional diversification (Figure 1). Similarly, the S-100B phylogenetic tree revealed two clusters. Group A included *Homo sapiens, Macaca mulatta*, and various rodents, indicating high functional conservation. Group B comprised a smaller but diverse set of species, highlighting the widespread distribution of S-100B across taxa (Figure 2). For UCH-L1, a highly conserved pattern was observed. Group A encompassed vertebrates such as *Mesocricetus auratus, Peromyscus maniculatus bairdii*, and *Homo sapiens*, underscoring its essential role in cellular processes. Notably, *Homo sapiens* clustered closely with *Macaca fascicularis*, reaffirming the evolutionary stability of UCH-L1 within primates. Group B, though smaller, demonstrated the presence of UCH-L1 across diverse species, reinforcing its fundamental biological importance (Figure 3).

**Table 2:** Maximum likelihood estimate of substitution matrix of GFAP protein of traumatic brain injury using MEGA 11

From \ To	A	R	N	D	C	Q	E	G	H	I	L	K	M	F	P	S	T	W	Y	V
**A**	-	0.1407	0.1230	0.2198	0.0604	0.1185	0.3417	0.6737	0.0262	0.0985	0.1464	0.1139	0.0570	0.0290	0.5131	1.3742	1.3896	0.0063	0.0234	1.0058
**R**	0.2117	-	0.0994	0.0411	0.1071	0.6428	0.1020	0.5262	0.3822	0.0651	0.1757	2.0124	0.0523	0.0137	0.1860	0.3540	0.1971	0.0934	0.0394	0.0591
**N**	0.2226	0.1195	-	1.4767	0.0330	0.1638	0.1855	0.2999	0.4802	0.1340	0.0649	0.7811	0.0402	0.0155	0.0319	1.7910	0.7141	0.0021	0.1175	0.0567
**D**	0.3295	0.0410	1.2234	-	0.0111	0.1110	2.4877	0.4926	0.1229	0.0316	0.0290	0.0871	0.0231	0.0068	0.0333	0.2083	0.1289	0.0043	0.0760	0.1084
**C**	0.2287	0.2697	0.0690	0.0280	-	0.0194	0.0173	0.2114	0.0863	0.0410	0.0777	0.0151	0.0496	0.1424	0.0324	0.7616	0.1424	0.0820	0.3538	0.2136
**Q**	0.2216	0.7992	0.1694	0.1385	0.0096	-	1.0944	0.0895	0.6766	0.0213	0.3346	0.9143	0.0554	0.0096	0.4209	0.1939	0.1588	0.0128	0.0426	0.0618
**E**	0.4252	0.0843	0.1276	2.0650	0.0057	0.7278	-	0.4323	0.0291	0.0305	0.0461	0.5343	0.0213	0.0092	0.0503	0.1106	0.1006	0.0085	0.0106	0.1602
**G**	0.6936	0.3600	0.1706	0.3383	0.0575	0.0492	0.3576	-	0.0240	0.0147	0.0328	0.0833	0.0158	0.0106	0.0545	0.6631	0.0962	0.0405	0.0088	0.1618
**H**	0.0876	0.8493	0.8873	0.2742	0.0762	1.2091	0.0781	0.0781	-	0.0495	0.2552	0.1619	0.0400	0.0952	0.2990	0.2628	0.1447	0.0095	0.9787	0.0419
**I**	0.1440	0.0633	0.1082	0.0308	0.0158	0.0167	0.0358	0.0208	0.0216	-	1.1024	0.0624	0.5862	0.1632	0.0258	0.1432	0.7743	0.0100	0.0508	3.2788
**L**	0.1236	0.0986	0.0303	0.0163	0.0173	0.1510	0.0312	0.0269	0.0644	0.6365	-	0.0452	0.4682	0.5254	0.2779	0.2096	0.0827	0.0394	0.0404	0.6062
**K**	0.1472	1.7283	0.5579	0.0751	0.0052	0.6315	0.5550	0.1045	0.0626	0.0552	0.0692	-	0.0758	0.0052	0.0567	0.1678	0.2930	0.0066	0.0147	0.0427
**M**	0.1872	0.1142	0.0730	0.0505	0.0430	0.0973	0.0561	0.0505	0.0393	1.3176	1.8229	0.1928	-	0.0917	0.0430	0.1011	0.6420	0.0150	0.0318	1.0462
**F**	0.0551	0.0173	0.0162	0.0087	0.0714	0.0097	0.0141	0.0195	0.0541	0.2119	1.1819	0.0076	0.0530	-	0.0389	0.3341	0.0422	0.0400	0.9192	0.2044
**P**	0.7814	0.1882	0.0269	0.0338	0.0130	0.3426	0.0616	0.0807	0.1362	0.0269	0.5013	0.0668	0.0199	0.0312	-	0.9869	0.3573	0.0052	0.0191	0.0728
**S**	1.5495	0.2652	1.1161	0.1567	0.2267	0.1169	0.1002	0.7263	0.0886	0.1105	0.2800	0.1464	0.0347	0.1984	0.7308	-	1.4500	0.0231	0.1053	0.1406
**T**	1.8267	0.1722	0.5188	0.1130	0.0494	0.1115	0.1063	0.1228	0.0569	0.6962	0.1288	0.2980	0.2568	0.0292	0.3084	1.6904	-	0.0060	0.0337	0.3938
**W**	0.0337	0.3338	0.0061	0.0153	0.1164	0.0368	0.0368	0.2113	0.0153	0.0368	0.2511	0.0276	0.0245	0.1133	0.0184	0.1103	0.0245	-	0.1256	0.0827
**Y**	0.0556	0.0624	0.1546	0.1207	0.2224	0.0542	0.0203	0.0203	0.6969	0.0827	0.1139	0.0271	0.0231	1.1525	0.0298	0.2224	0.0610	0.0556	-	0.0569
**V**	1.1648	0.0455	0.0363	0.0838	0.0653	0.0383	0.1491	0.1820	0.0145	2.5974	0.8317	0.0383	0.3687	0.1247	0.0554	0.1444	0.3469	0.0178	0.0277	-

Each entry is the probability of substitution (r) from one amino acid (row) to another (column). Substitution patterns and rates were estimated under the Jones-Taylor-Thornton model (Jones et al. 1992). Relative values of instantaneous r should be considered when evaluating them. For simplicity, the sum of r values is made equal to 100. The amino acid frequencies are 7.69% (A), 5.11% (R), 4.25% (N), 5.13% (D), 2.03% (C), 4.11% (Q), 6.18% (E), 7.47% (G), 2.30% (H), 5.26% (I), 9.11% (L), 5.95% (K), 2.34% (M), 4.05% (F), 5.05% (P), 6.82% (S), 5.85% (T), 1.43% (W), 3.23% (Y), and 6.64% (V). For estimating ML values, a tree topology was automatically computed. The maximum Log-likelihood for this computation was –2493.154. This analysis involved 42 amino acid sequences. There was a total of 436 positions in the final dataset. Evolutionary analyses were conducted in MEGA11 (Tamura et al. 2021)

**Table 3:** Maximum likelihood estimate of substitution matrix of S-100B protein of traumatic brain injury using MEGA 11

From \ To	A	R	N	D	C	Q	E	G	H	I	L	K	M	F	P	S	T	W	Y	V
**A**	–	0.1407	0.1230	0.2198	0.0604	0.1185	0.3417	0.6737	0.0262	0.0985	0.1464	0.1139	0.0570	0.0290	0.5131	1.3742	1.3896	0.0063	0.0234	1.0058
**R**	0.2117	–	0.0994	0.0411	0.1071	0.6428	0.1020	0.5262	0.3822	0.0651	0.1757	2.0124	0.0523	0.0137	0.1860	0.3540	0.1971	0.0934	0.0394	0.0591
**N**	0.2226	0.1195	–	1.4767	0.0330	0.1638	0.1855	0.2999	0.4802	0.1340	0.0649	0.7811	0.0402	0.0155	0.0319	1.7910	0.7141	0.0021	0.1175	0.0567
**D**	0.3295	0.0410	1.2234	–	0.0111	0.1110	2.4877	0.4926	0.1229	0.0316	0.0290	0.0871	0.0231	0.0068	0.0333	0.2083	0.1289	0.0043	0.0760	0.1084
**C**	0.2287	0.2697	0.0690	0.0280	–	0.0194	0.0173	0.2114	0.0863	0.0410	0.0777	0.0151	0.0496	0.1424	0.0324	0.7616	0.1424	0.0820	0.3538	0.2136
**Q**	0.2216	0.7992	0.1694	0.1385	0.0096	–	1.0944	0.0895	0.6766	0.0213	0.3346	0.9143	0.0554	0.0096	0.4209	0.1939	0.1588	0.0128	0.0426	0.0618
**E**	0.4252	0.0843	0.1276	2.0650	0.0057	0.7278	–	0.4323	0.0291	0.0305	0.0461	0.5343	0.0213	0.0092	0.0503	0.1106	0.1006	0.0085	0.0106	0.1602
**G**	0.6936	0.3600	0.1706	0.3383	0.0575	0.0492	0.3576	–	0.0240	0.0147	0.0328	0.0833	0.0158	0.0106	0.0545	0.6631	0.0962	0.0405	0.0088	0.1618
**H**	0.0876	0.8493	0.8873	0.2742	0.0762	1.2091	0.0781	0.0781	–	0.0495	0.2552	0.1619	0.0400	0.0952	0.2990	0.2628	0.1447	0.0095	0.9787	0.0419
**I**	0.1440	0.0633	0.1082	0.0308	0.0158	0.0167	0.0358	0.0208	0.0216	–	1.1024	0.0624	0.5862	0.1632	0.0258	0.1432	0.7743	0.0100	0.0508	3.2788
**L**	0.1236	0.0986	0.0303	0.0163	0.0173	0.1510	0.0312	0.0269	0.0644	0.6365	–	0.0452	0.4682	0.5254	0.2779	0.2096	0.0827	0.0394	0.0404	0.6062
**K**	0.1472	1.7283	0.5579	0.0751	0.0052	0.6315	0.5550	0.1045	0.0626	0.0552	0.0692	–	0.0758	0.0052	0.0567	0.1678	0.2930	0.0066	0.0147	0.0427
**M**	0.1872	0.1142	0.0730	0.0505	0.0430	0.0973	0.0561	0.0505	0.0393	1.3176	1.8229	0.1928	–	0.0917	0.0430	0.1011	0.6420	0.0150	0.0318	1.0462
**F**	0.0551	0.0173	0.0162	0.0087	0.0714	0.0097	0.0141	0.0195	0.0541	0.2119	1.1819	0.0076	0.0530	–	0.0389	0.3341	0.0422	0.0400	0.9192	0.2044
**P**	0.7814	0.1882	0.0269	0.0338	0.0130	0.3426	0.0616	0.0807	0.1362	0.0269	0.5013	0.0668	0.0199	0.0312	–	0.9869	0.3573	0.0052	0.0191	0.0728
**S**	1.5495	0.2652	1.1161	0.1567	0.2267	0.1169	0.1002	0.7263	0.0886	0.1105	0.2800	0.1464	0.0347	0.1984	0.7308	–	1.4500	0.0231	0.1053	0.1406
**T**	1.8267	0.1722	0.5188	0.1130	0.0494	0.1115	0.1063	0.1228	0.0569	0.6962	0.1288	0.2980	0.2568	0.0292	0.3084	1.6904	–	0.0060	0.0337	0.3938
**W**	0.0337	0.3338	0.0061	0.0153	0.1164	0.0368	0.0368	0.2113	0.0153	0.0368	0.2511	0.0276	0.0245	0.1133	0.0184	0.1103	0.0245	–	0.1256	0.0827
**Y**	0.0556	0.0624	0.1546	0.1207	0.2224	0.0542	0.0203	0.0203	0.6969	0.0827	0.1139	0.0271	0.0231	1.1525	0.0298	0.2224	0.0610	0.0556	–	0.0569
**V**	1.1648	0.0455	0.0363	0.0838	0.0653	0.0383	0.1491	0.1820	0.0145	2.5974	0.8317	0.0383	0.3687	0.1247	0.0554	0.1444	0.3469	0.0178	0.0277	–

Each entry is the probability of substitution (r) from one amino acid (row) to another (column). Substitution patterns and rates were estimated under the Jones-Taylor-Thornton model (Jones et al. 1992). Relative values of instantaneous r should be considered when evaluating them. For simplicity, the sum of r values is made equal to 100. The amino acid frequencies are 7.69% (A), 5.11% (R), 4.25% (N), 5.13% (D), 2.03% (C), 4.11% (Q), 6.18% (E), 7.47% (G), 2.30% (H), 5.26% (I), 9.11% (L), 5.95% (K), 2.34% (M), 4.05% (F), 5.05% (P), 6.82% (S), 5.85% (T), 1.43% (W), 3.23% (Y), and 6.64% (V). For estimating ML values, a tree topology was automatically computed. The maximum log-likelihood for this computation was –516.110. This analysis involved 42 amino acid sequences. There was a total of 436 positions in the final dataset. Evolutionary analyses were conducted in MEGA11 (Tamura et al. 2021)

**Table 4:** Maximum likelihood estimate of substitution matrix of UCH-L1 protein of traumatic brain injury using MEGA 11

From \ To	A	R	N	D	C	Q	E	G	H	I	L	K	M	F	P	S	T	W	Y	V
**A**	–	0.1407	0.1230	0.2198	0.0604	0.1185	0.3417	0.6737	0.0262	0.0985	0.1464	0.1139	0.0570	0.0290	0.5131	1.3742	1.3896	0.0063	0.0234	1.0058
**R**	0.2117	–	0.0994	0.0411	0.1071	0.6428	0.1020	0.5262	0.3822	0.0651	0.1757	2.0124	0.0523	0.0137	0.1860	0.3540	0.1971	0.0934	0.0394	0.0591
**N**	0.2226	0.1195	–	1.4767	0.0330	0.1638	0.1855	0.2999	0.4802	0.1340	0.0649	0.7811	0.0402	0.0155	0.0319	1.7910	0.7141	0.0021	0.1175	0.0567
**D**	0.3295	0.0410	1.2234	–	0.0111	0.1110	2.4877	0.4926	0.1229	0.0316	0.0290	0.0871	0.0231	0.0068	0.0333	0.2083	0.1289	0.0043	0.0760	0.1084
**C**	0.2287	0.2697	0.0690	0.0280	–	0.0194	0.0173	0.2114	0.0863	0.0410	0.0777	0.0151	0.0496	0.1424	0.0324	0.7616	0.1424	0.0820	0.3538	0.2136
**Q**	0.2216	0.7992	0.1694	0.1385	0.0096	–	1.0944	0.0895	0.6766	0.0213	0.3346	0.9143	0.0554	0.0096	0.4209	0.1939	0.1588	0.0128	0.0426	0.0618
**E**	0.4252	0.0843	0.1276	2.0650	0.0057	0.7278	–	0.4323	0.0291	0.0305	0.0461	0.5343	0.0213	0.0092	0.0503	0.1106	0.1006	0.0085	0.0106	0.1602
**G**	0.6936	0.3600	0.1706	0.3383	0.0575	0.0492	0.3576	–	0.0240	0.0147	0.0328	0.0833	0.0158	0.0106	0.0545	0.6631	0.0962	0.0405	0.0088	0.1618
**H**	0.0876	0.8493	0.8873	0.2742	0.0762	1.2091	0.0781	0.0781	–	0.0495	0.2552	0.1619	0.0400	0.0952	0.2990	0.2628	0.1447	0.0095	0.9787	0.0419
**I**	0.1440	0.0633	0.1082	0.0308	0.0158	0.0167	0.0358	0.0208	0.0216	–	1.1024	0.0624	0.5862	0.1632	0.0258	0.1432	0.7743	0.0100	0.0508	3.2788
**L**	0.1236	0.0986	0.0303	0.0163	0.0173	0.1510	0.0312	0.0269	0.0644	0.6365	–	0.0452	0.4682	0.5254	0.2779	0.2096	0.0827	0.0394	0.0404	0.6062
**K**	0.1472	1.7283	0.5579	0.0751	0.0052	0.6315	0.5550	0.1045	0.0626	0.0552	0.0692	–	0.0758	0.0052	0.0567	0.1678	0.2930	0.0066	0.0147	0.0427
**M**	0.1872	0.1142	0.0730	0.0505	0.0430	0.0973	0.0561	0.0505	0.0393	1.3176	1.8229	0.1928	–	0.0917	0.0430	0.1011	0.6420	0.0150	0.0318	1.0462
**F**	0.0551	0.0173	0.0162	0.0087	0.0714	0.0097	0.0141	0.0195	0.0541	0.2119	1.1819	0.0076	0.0530	–	0.0389	0.3341	0.0422	0.0400	0.9192	0.2044
**P**	0.7814	0.1882	0.0269	0.0338	0.0130	0.3426	0.0616	0.0807	0.1362	0.0269	0.5013	0.0668	0.0199	0.0312	–	0.9869	0.3573	0.0052	0.0191	0.0728
**S**	1.5495	0.2652	1.1161	0.1567	0.2267	0.1169	0.1002	0.7263	0.0886	0.1105	0.2800	0.1464	0.0347	0.1984	0.7308	–	1.4500	0.0231	0.1053	0.1406
**T**	1.8267	0.1722	0.5188	0.1130	0.0494	0.1115	0.1063	0.1228	0.0569	0.6962	0.1288	0.2980	0.2568	0.0292	0.3084	1.6904	–	0.0060	0.0337	0.3938
**W**	0.0337	0.3338	0.0061	0.0153	0.1164	0.0368	0.0368	0.2113	0.0153	0.0368	0.2511	0.0276	0.0245	0.1133	0.0184	0.1103	0.0245	–	0.1256	0.0827
**Y**	0.0556	0.0624	0.1546	0.1207	0.2224	0.0542	0.0203	0.0203	0.6969	0.0827	0.1139	0.0271	0.0231	1.1525	0.0298	0.2224	0.0610	0.0556	–	0.0569
**V**	1.1648	0.0455	0.0363	0.0838	0.0653	0.0383	0.1491	0.1820	0.0145	2.5974	0.8317	0.0383	0.3687	0.1247	0.0554	0.1444	0.3469	0.0178	0.0277	–

Each entry is the probability of substitution (r) from one amino acid (row) to another (column). Substitution pattern and rates were estimated under the Jones-Taylor-Thornton model (Jones et al. 1992). Relative values of instantaneous r should be considered when evaluating them. For simplicity, the sum of r values is made equal to 100. The amino acid frequencies are 7.69% (A), 5.11% (R), 4.25% (N), 5.13% (D), 2.03% (C), 4.11% (Q), 6.18% (E), 7.47% (G), 2.30% (H), 5.26% (I), 9.11% (L), 5.95% (K), 2.34% (M), 4.05% (F), 5.05% (P), 6.82% (S), 5.85% (T), 1.43% (W), 3.23% (Y), and 6.64% (V). For estimating ML values, a tree topology was automatically computed. The maximum log-likelihood for this computation was –1195.037. This analysis involved 42 amino acid sequences. There was a total of 223 positions in the final dataset. Evolutionary analyses were conducted in MEGA11 (Tamura et al. 2021). Click or tap here to enter text

## Domain separation

**Figure 4:**
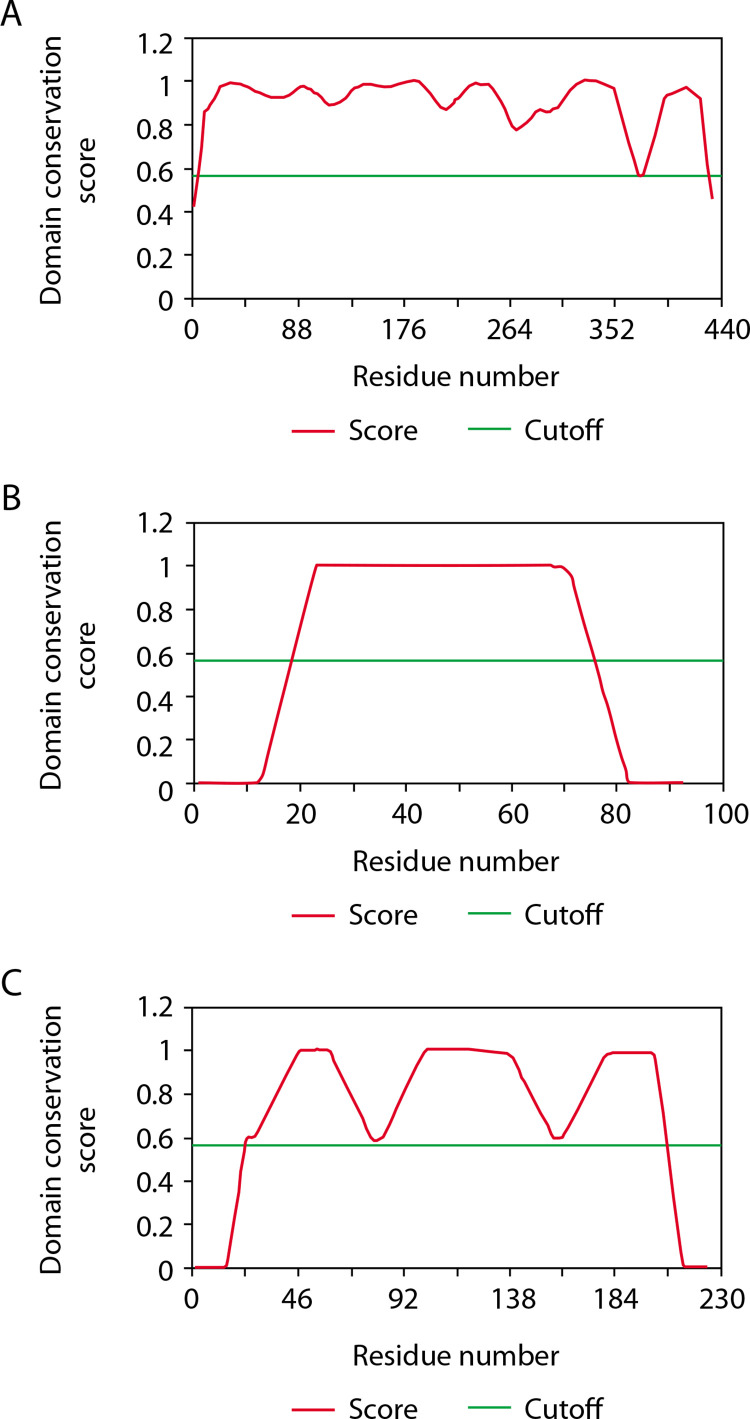
Domain separation of (**A**) the GFAP protein (T11613), (**B**) the S-100B protein (T11612), and (**C**) the UCH-L1 protein (T11624) using the ThreaDom server

The CD-Search results provide domain multiple sequence alignments by integrating user queries and annotating protein domains on these sequences. For the GFAP protein, the NCBI Conserved Domain Search identified two domains: one with accession number pfam00038, spanning intervals 68–376 with an *E*-value of 1.12e–127, and another with accession number pfam04732, covering intervals 4–66 with an *E*-value of 2.51e–08. ThreaDom analysis also revealed two domains for GFAP, spanning 1–171 and 172–345, with a cutoff of 0.56. Similarly, the S-100B protein showed one domain via NCBI CDD, with accession number cd05027, an interval of 2–89, and an *E*-value of 1.68e–47. ThreaDom analysis identified a single domain in GFAP with the same cutoff of 0.56. For the UCH-L1 protein, NCBI CDD revealed a single domain with accession number cd09616, spanning intervals 5–219 with an *E*-value of 3.16e–127. ThreaDom also identified one domain in GFAP, using the same cutoff of 0.56 (Table 5 and Figure 4).

**Table 5:** Domain assignment of GFAP, S-100B and UCH-L1 protein using CD-Search (NCBI server)

Proteins^a^	Name^b^	Accession^c^	Description^d^	Interval^e^	*E* -value^f^	Bitscore^g^	Superfamily^h^
GFAP	Filament	pfam00038	Intermediate filament protein	68–376	1.12e–127	371.555	cl25641
	Filament_head	pfam04732	Intermediate filament head (DNA binding) region: This family represents the N-terminal head…	4–66	2.51e–08	50.8519	cl04711
S-100B	S-100B	cd05027	S-100B: The S-100B domain is found in proteins similar to S100B. S100B is a calciumbinding protein	2–89	1.68e–47	146.155	cl08302
UCH-L1	Peptidase_C12_ UCH_L1_L3	cd09616	Cysteine peptidase C12 containing ubiquitin carboxyl-terminal hydrolase ( UCH) families L1 and…	5–219	3.16e–127	321.122	cl08306

a Proteins: The analyzed protein names.

b Name: The specific domain or structural component of the protein.

c Accession: The unique identifier assigned to the protein family or domain in the database.

d Description: A brief functional or structural description of the protein or domain.

e Interval: The residue range within the protein where the domain is located.

f E-value: The statistical significance of the match, with lower values indicating higher confidence.

g Bitscore: A sequence similarity measure where higher scores indicate more decisive matches.

h Superfamily: The broader classification of structurally and functionally related proteins

## Secondary structure and solvent accessibility prediction

The GFAP protein’s secondary structure is predominantly alpha-helical, accounting for 65% (281 residues), with a minor presence of beta strands (6%, 25 residues) and coils (28%, 120 residues), achieving an overall prediction confidence of 86%. Similarly, the S-100B protein is helix-dominant, with 67% (63 residues) forming alpha helices, no beta strands, and 33% (19 residues) structured as coils, with an 87.5% confidence level. In contrast, UCH-L1 exhibits a more balanced composition, with alpha helices and coils each constituting 41% (91 and 92 residues, respectively), while beta strands make up 18% (40 residues), with an 80.4% confidence level.

Solvent accessibility analyses indicate that GFAP and S-100B are primarily buried, with solvent exposure levels of 63.89% and 66.30%, respectively, while UCH-L1 has a more exposed surface, with 42.60% solvent-accessible regions compared to 57.40% buried regions. These structural characteristics provide valuable insights into the proteins’ solvent interactions and potential functional dynamics (Table 6). This concise overview is suitable for inclusion in a review article, offering a clear snapshot of the proteins’ structural profiles.

**Table 6:** Predicted secondary structure of proteins using different servers

Protein^a^	2ry structure^b^			Exposed^c^	Intermediate^d^	Buried^e^
	Alpha helix	Beta sheet	Others (Coil-Turn-Loop)			
GFAP	65%	6.2%	28.7%	36.11%	–	63.89 %
S-100B	67.39%	0%	32.61%	33.70%	–	66.30 %
UCH-L1	40.81%	17.94%	41.26%	42.60%	–	57.40 %

a Protein: The analyzed protein name.

b 2ry structure (secondary structure): The predicted composition of the protein secondary structure elements.

c Exposed: The percentage of residues that are solvent-exposed on the protein surface.

d Intermediate: The percentage of residues partially buried in the protein structure.

e Buried: The percentage of residues fully buried within the protein core

## Three-dimensional (3-D) structure prediction

Initial models were generated, developed, and reviewed using several servers aligned with CASP15 protocols to create the 3D model, and the highest-quality model was selected.

### Construction of an initial model using target-template alignment

GalaxyWEB, Swiss-Model, and LOMETS were used for aligned regions, while I-TASSER, Robetta, Phyre2, and AlphaFold targeted low-similarity regions to construct structural models for unaligned regions. AlphaFold demonstrated superior performance, particularly in modeling full-length structures with high confidence scores, making it a critical tool for assessing structural integrity.

For the GFAP protein, I-TASSER generated five models, with a C-score of –3.23 for the main protein, –3.24 for Domain 1, and –1.15 for Domain 2. In contrast, AlphaFold provided a QMEAN Z-score of 0.89, indicating a highly accurate model. In the case of the S-100B protein, the C-scores were 0.06 for the main protein, –0.5 for Domain 1, and –0.25 for Domain 2, while AlphaFold achieved a QMEAN score of 0.79 ± 0.09, confirming its reliability. For the UCH-L1 protein, I-TASSER developed five models, with a C-score of 1.51 for Domain 1. In contrast, AlphaFold provided an RMSD value of 3.36 Å after refinement, suggesting enhanced accuracy in secondary structure alignment.

Each query sequence was given five models by GalaxyWEB, which also selected templates for modeling by rescoring HHsearch results. While Phyre2 built 3D models using advanced distant homology detection techniques, SWISS-MODEL generated multiple models with QMEAN scores of 0.86 ± 0.06, 0.27 ± 0.12, and 0.69 ± 0.07 for GFAP; 0.81 ± 0.06, 0.80 ± 0.09, and 0.81 ± 0.11 for S-100B; and 0.86 ± 0.06 and 0.87 ± 0.06 for UCH-L1. Among these, AlphaFold consistently ranked as one of the top-performing predictors, producing models with high structural fidelity across all three biomarkers.

### Reduced-level structure assembly and refinement simulations

The second stage of structure prediction involved refining the S-100B protein. In terms of hydrogen bonds, backbone structure, and side-chain positioning, the results from the GalaxyWEB, ModRefiner, and 3Drefine servers successfully optimized the basic starting models, bringing them closer to their native state. Refinement improved the physical quality of global and local structures compared to the original model generated by selected servers, such as I-TASSER for the target domains. This was achieved by lowering the RMSD and clash scores while increasing the TM-score, enhancing structural accuracy and stability.

## Model evaluation and selection

The best 3D model of the correct fold was chosen through model evaluation from all generated conformations, selecting those most closely resembling the native structure. Various evaluation metrics were used to assess structural accuracy and stability, including Swiss-Model Works, QMEAN Server, TM-align, TM-score, Z-score, RMSD, Clash-score, and PROCHECK. AlphaFold and I-TASSER were identified as the best-performing approaches, consistently ranking among the top predictors in CASP11, CASP12, CASP13, CASP14, and CASP15 assessments.

The I-TASSER server produced five full-length models with high C-scores, an estimated TM-score of 0.92 ± 0.06, and an RMSD of 2.7 ± 2.0 Å, confirming the accuracy of its models. However, AlphaFold delivered the best structural predictions for GFAP, S-100B, and UCH-L1, with TM-scores exceeding 0.99, demonstrating near-native accuracy. The selected AlphaFold models outperformed other methods in terms of RMSD reduction and global alignment accuracy, making them the optimal choice for further structural and functional interpretation.

The LOMETS server’s best prediction of the threedimensional structures of GFAP, S-100B, and UCH-L1 (Table 10) further validated AlphaFold’s superiority. The estimated scores for the projected three-dimensional structures using AlphaFold consistently ranked higher than experimentally determined structures in terms of RMSD, TM-score, *C*-score, QMEAN *Z*-score, MolProbity score, and Clash score (Tables 7–10).

**Table 7:** Three-dimensional structure prediction of the GFAP protein for the main protein

Servers^a^ \ Scores	I-Tasser	Lomets	Robetta	Phyre2	Swiss-Model	AlphaFold
RMSD^b^						
3Drefine	3	3.2	2.68	2.7	1.99	2.83
GalaxyWebrefine	2.97	3.18	2.86	2.5	2.19	2.89
Modrefine	3.07	3.29	3.41	2.62	2.01	3.08
DeepRefiner	0.58	3.26	3.49	2.87	2.6	2.54
TM-score^c^						
3Drefine	0.9655	0.9338	0.6641	0.9748	0.8489	0.7870
GalaxyWebrefine	0.9900	0.9483	0.9112	0.9900	0.9740	0.8215
Modrefine	0.9975	0.7093	0.9671	0.9993	0.9918	0.9449
DeepRefiner	0.9854	0.9216	0.8815	0.9663	0.9461	0.8796
GDT-TS^d^						
3Drefine	0.1852	0.1869	0.2182	0.6538	0.4903	0.1794
GalaxyWebrefine	0.1892	0.1858	0.2164	0.6593	0.4854	0.173
Modrefine	0.1794	0.184	0.2153	0.6758	0.5211	0.1649
DeepRefiner	0.2582	0.1874	0.1887	0.7198	0.4984	0.1719
GDT-HA^e^						
3Drefine	0.1146	0.1325	0.1476	0.4643	0.3312	0.1285
GalaxyWebrefine	0.1192	0.1308	0.1481	0.4643	0.3231	0.1273
Modrefine	0.1076	0.1267	0.1447	0.4918	0.362	0.1152
DeepRefiner	0.2228	0.1276	0.1238	0.5247	0.3312	0.1238
QMEAN^f^						
3Drefine	0.51 ± 0.05	0.53 ± 0.05	0.57 ± 0.05	0.74 ± 0.09	0.70 ± 0.07	0.58 ± 0.05
GalaxyWebrefine	0.52 ± 0.05	0.52 ± 0.05	0.57 ± 0.05	0.77 ± 0.09	0.73 ± 0.07	0.60 ± 0.05
Modrefine	0.51 ± 0.05	0.52 ± 0.05	0.55 ± 0.05	0.77 ± 0.09	0.73 ± 0.07	0.57 ± 0.05
DeepRefiner	0.73 ± 0.09	0.54 ± 0.05	0.58 ± 0.05	0.76 ± 0.09	0.73 ± 0.07	0.59 ± 0.05
MolProbity^g^						
3Drefine	3.73	1.9	1.29	1.65	1.39	2.04
GalaxyWebrefine	2.33	1.33	0.73	0.8	1.03	0.69
Modrefine	2.56	2.18	1.52	1.37	1.46	1.59
DeepRefiner	2.73	2.95	2.61	2.62	2.74	3.1
Clash score^h^						
3Drefine	40.18	6.14	2.57	13.95	7.06	6
GalaxyWebrefine	13.28	2.71	0.71	0.66	1.57	0.57
Modrefine	40.86	25	9.86	6.65	8.63	11.29
DeepRefiner	185.48	157.7	130.93	143.73	126.97	129.11
Aligned length^i^	156	199	184	89	127	151
RF^j^	84.19%	96.05%	99.07%	100%	98.68%	99.07 %
Overall factor^k^	86.32%	95.88%	-	100%	100%	99.70 %

a Servers: The computational protein structure prediction and refinement tools.

b RMSD (root mean square deviation): Measures the average deviation between the predicted and reference structures, with lower values indicating better accuracy.

c TM**-**score (template modeling score): Assesses the similarity between the predicted and native structures, where values closer to 1 indicate higher accuracy.

d GDT-TS (Global Distance Test-Total Score): Evaluates the accuracy of structural alignment by considering the fraction of residues within a certain distance threshold from the reference structure.

e GDT-HA (Global Distance Test-High Accuracy): A more stringent version of GDT-TS, focusing on higher precision in structural alignment.

f QMEAN (Qualitative Model Energy Analysis): A composite score reflecting the overall quality of the predicted structure based on statistical potentials.

g MolProbity: A structural validation score considering atomic clashes, bond angles, and steric hindrances, where lower values indicate better quality.

h Clash score: The number of atomic clashes per 1000 atoms, with lower values suggesting fewer steric conflicts.

i Aligned length: The number of residues successfully aligned between the predicted and reference structures.

j RF (Residue Frequency): The percentage of correctly predicted residues compared to the reference structure.

k Overall factor: A combined score reflecting the overall reliability of the predicted model

**Table 8:** 3D-Structure prediction of S100B protein for the main protein

Servers^a^ \ Scores	I-Tasser	Lomets	Quark	Robetta	Phyre2	Swiss-Model	AlphaFold
RMSD^b^							
3Drefine	3.37	3.62	3.41	3.35	3.32	3.25	3.42
GalaxyWebrefine	3.41	3.63	3.39	3.39	3.33	3.25	3.44
Modrefine	3.28	3.37	2.93	3.29	3.27	3.33	3.4
DeepRefiner	3.45	3.62	3.54	3.44	3.6	3.38	3.38
TM-score^c^							
3Drefine	0.9874	0.9973	0.9951	0.9835	0.9874	0.9838	0.9905
GalaxyWebrefine	0.9876	0.9982	0.9966	0.9838	0.9693	0.9803	0.9886
Modrefine	0.9978	0.9982	0.9992	0.9998	0.9996	0.9999	0.9992
DeepRefiner	0.9807	0.9980	0.9925	0.9873	0.9850	0.4927	0.9892
GDT-TS^d^							
3Drefine	0.4484	0.4864	0.4239	0.4565	0.4185	0.4429	0.4429
GalaxyWebrefine	0.4511	0.4973	0.4321	0.4538	0.4266	0.4429	0.4484
Modrefine	0.4511	0.3886	0.074	0.4565	0.4348	0.4484	0.4457
DeepRefiner	0.4457	0.4725	0.4049	0.4592	0.4049	0.2199	0.4484
GDT-HA^e^							
3Drefine	0.2418	0.269	0.2337	0.25	0.2201	0.25	0.2446
GalaxyWebrefine	0.2391	0.2977	0.2418	0.2446	0.2283	0.25	0.25
Modrefine	0.2391	0.1957	0.0504	0.2554	0.2418	0.2527	0.2473
DeepRefiner	0.2364	0.2527	0.2092	0.25	0.212	0.123	0.25
QMEAN^f^							
3Drefine	0.76 ± 0.09	0.70 ± 0.09	0.69 ± 0.09	0.77 ± 0.09	0.66 ± 0.09	0.82 ± 0.06	0.80 ± 0.09
GalaxyWebrefine	0.74 ± 0.09	0.69 ± 0.09	0.71 ± 0.09	0.75 ± 0.09	0.68 ± 0.09	0.82 ± 0.06	0.79 ± 0.09
Modrefine	0.73 ± 0.09	0.40 ± 0.09	0.84 ± 0.06	0.76 ± 0.09	0.66 ± 0.09	0.78 ± 0.09	0.79 ± 0.09
DeepRefiner	0.75 ± 0.09	0.71 ± 0.09	0.66 ± 0.09	0.76 ± 0.09	0.68 ± 0.09	0.80 ± 0.06	0.79 ± 0.09
MolProbity^g^							
3Drefine	3.09	1.62	3.21	1.38	1.85	1.43	1.13
GalaxyWebrefine	1.46	1.5	1.75	1.51	0.92	1.43	0.72
Modrefine	2.47	2.86	2.18	2.26	1.87	1.84	1.93
DeepRefiner	2.99	2.66	2.69	2.68	2.65	2.76	2.67
Clash score^h^							
3Drefine	20.58	4.8	22.63	6.86	7.54	1.38	3.43
GalaxyWebrefine	4.8	3.43	7.54	8.23	1.37	1.38	0.69
Modrefine	45.3	56.97	47.92	37.06	21.28	21.96	27.45
DeepRefiner	182.03	160.05	171.66	164.17	153.23	185.93	160.71
Aligned length^i^	85	89	27	83	80	29	79
RF^j^	93.33%	95.56%	93.33%	98.89%	93.33%	100.00%	100.00 %
Overall factor^k^	100.00%	89.29%	97.62%	100.00%	96.34%	92%	100.00 %

a Servers: The computational protein structure prediction and refinement tools.

b RMSD (root mean square deviation): Measures the average deviation between the predicted and reference structures, with lower values indicating better accuracy.

c TM-score (template modeling score): Assesses the similarity between the predicted and native structures, where values closer to 1 indicate higher accuracy.

d GDT-TS (Global Distance Test-Total Score): Evaluates the accuracy of structural alignment by considering the fraction of residues within a certain distance threshold from the reference structure.

e GDT-HA (Global Distance Test-High Accuracy): A more stringent version of GDT-TS, focusing on higher precision in structural alignment.

f QMEAN (Qualitative Model Energy Analysis): A composite score reflecting the overall quality of the predicted structure based on statistical potentials.

g MolProbity: A structural validation score considering atomic clashes, bond angles, and steric hindrances, where lower values indicate better quality.

h Clash score: The number of atomic clashes per 1000 atoms, with lower values suggesting fewer steric conflicts.

i Aligned length: The number of residues successfully aligned between the predicted and reference structures.

j RF (residue frequency): The percentage of correctly predicted residues compared to the reference structure.

k Overall factor: A combined score reflecting the overall reliability of the predicted model

**Table 9:** 3D-Structure prediction of UCH-L1 protein for the main protein

Servers^a^ \ Scores	I-Tasser	Lomets	Robetta	Phyre2	Swiss-Model	AlphaFold
RMSD^b^						
3Drefine	3.21	2.93	3.08	3.21	3.27	3.36
GalaxyWebrefine	3.46	2.86	3.12	3.23	3.27	3.34
Modrefine	3.21	2.93	3.17	3.2	3.35	3.36
DeepRefiner	3.33	3	3.1	3.34	3.27	3.25
TM-score^c^						
3Drefine	0.9958	0.9965	0.9966	0.9966	0.9923	0.9963
GalaxyWebrefine	0.9960	0.9964	0.9975	0.9956	0.9960	0.9956
Modrefine	0.9996	0.9988	0.9994	0.9991	0.9993	0.9997
DeepRefiner	0.9968	0.9949	0.9932	0.9891	0.7443	0.9946
GDT-TS^d^						
3Drefine	0.093	0.0953	0.0919	0.0919	0.0942	0.0897
GalaxyWebrefine	0.0942	0.0953	0.0942	0.093	0.0942	0.0919
Modrefine	0.0942	0.0953	0.0953	0.093	0.0942	0.0908
DeepRefiner	0.0908	0.0968	0.0942	0.093	0.0942	0.0886
GDT-HA^e^						
3Drefine	0.0493	0.0516	0.0482	0.0482	0.0516	0.0493
GalaxyWebrefine	0.0504	0.0516	0.0504	0.0482	0.0516	0.0504
Modrefine	0.0504	0.0504	0.0493	0.0482	0.0493	0.0493
DeepRefiner	0.046	0.0518	0.0493	0.046	0.0516	0.046
QMEAN^f^						
3Drefine	0.88 ± 0.06	0.82 ± 0.06	0.81 ± 0.06	0.86 ± 0.06	0.87 ± 0.06	0.86 ± 0.06
GalaxyWebrefine	0.86 ± 0.06	0.81 ± 0.06	0.81 ± 0.06	0.87 ± 0.06	0.87 ± 0.06	0.86 ± 0.06
Modrefine	0.84 ± 0.06	0.80 ± 0.06	0.79 ± 0.06	0.85 ± 0.06	0.86 ± 0.06	0.84 ± 0.06
DeepRefiner	0.83 ± 0.06	0.87 ± 0.06	0.81 ± 0.06	0.85 ± 0.06	0.87 ± 0.06	0.85 ± 0.06
MolProbity^g^						
3Drefine	2.74	1.66	1.56	1.76	1.14	1.76
GalaxyWebrefine	1.3	1.62	1.47	1.18	1.14	1.29
Modrefine	2.11	2.36	2.16	2.09	2.25	2.25
DeepRefiner	2.95	2.96	2.87	2.89	1.14	2.71
Clash score^h^						
3Drefine	13.28	6.35	5.19	17.32	1.7	5.48
GalaxyWebrefine	5.48	6.93	7.5	3.75	1.7	3.46
Modrefine	43.3	43.3	47.92	38.39	41.28	40.99
DeepRefiner	188.54	192.07	190.72	200.63	1.7	176.94
Aligned length^i^	79	89	87	85	91	91
RF^j^	93.67%	95.48%	95.93%	98.19%	96.83%	100.00 %
Overall factor^k^	94.88%	83.57%	91.63%	94%	98.09%	92.99 %

a Servers: The computational protein structure prediction and refinement tools.

b RMSD (root mean square deviation): Measures the average deviation between the predicted and reference structures, with lower values indicating better accuracy.

c TM-score (template modeling score): Assesses the similarity between the predicted and native structures, where values closer to 1 indicate higher accuracy.

d GDT-TS (Global Distance Test-Total Score): Evaluates the accuracy of structural alignment by considering the fraction of residues within a certain distance threshold from the reference structure.

e GDT-HA (Global Distance Test-High Accuracy): A more stringent version of GDT-TS, focusing on higher precision in structural alignment.

f QMEAN (Qualitative Model Energy Analysis): A composite score reflecting the overall quality of the predicted structure based on statistical potentials.

g MolProbity: A structural validation score considering atomic clashes, bond angles, and steric hindrances, where lower values indicate better quality.

h Clash score: The number of atomic clashes per 1000 atoms, with lower values suggesting fewer steric conflicts.

i Aligned length: The number of residues successfully aligned between the predicted and reference structures.

j RF (residue frequency): The percentage of correctly predicted residues compared to the reference structure.

k Overall factor:A combined score reflecting the overall reliability of the predicted model

**Table 10:** Best template structure for GFAP, S-100B, and UCH-L1

Protein^a^	Subject	Tm-Score	RMSD^b^	Sequence identity	Cov^c^
GFAP	7ogtB1	0.67	1.17	0.104	0.683
S-100B	1xk4L	0.947	0.64	0.378	0.978
UCH-L1	2etlA	0.994	0.42	1	1

a Protein ranking is based on the TM-score of the structural alignment between the query structure and known structures in the PDB library.

b RMSDa is the RMSD between residues that TM-align structurally aligns.

c Cov represents the coverage of the alignment by TM-align and is equal to the number of structurally aligned residues divided by the length of the query protein

### Motifs prediction

#### Motif analysis

**Figure 5:**
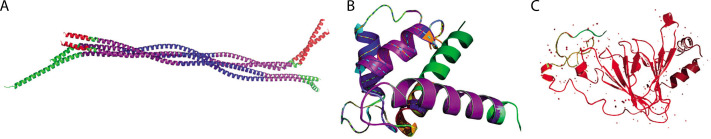
**A**) Cartoon representation of the GFAP protein shows known and predicted motifs, where the Filament (68–376) is highlighted in purple, Filament_head (7–66) in red, DUF1664 (129–199) in blue, and DUF1664_2 (226–315) in green. **B**) The cartoon view showed known and predicted motifs of S-100B, where S_100 (4–47) is highlighted in purple, EF-hand_1 in red, EF-hand_7 in blue, EF-hand_6 in green, EF-hand_4 in orange, EF-hand_5 in brown, EF-hand_8 in yellow, and Spt20 in cyan. **C**) The cartoon view showed known and predicted motifs of UCH-L1 protein is displayed with the (3–221) region highlighted in red, while the remaining structure is in green

Utilizing MotifFinder and MotifScan, we analyzed motifs in GFAP, S-100B, and UCH-L1 proteins to uncover sequence patterns associated with specific functions. These tools, employing distinct algorithms, provide a comprehensive view of conserved motifs within these proteins. Motif analysis of GFAP, S-100B, and UCH-L1 using MotifFinder and MotifScan servers revealed critical insights. GFAP’s motifs, including Filament and Filament_head, serve as structural foundations for astrocytic integrity, while unknown motifs suggest potential novel functions. S-100B’s calcium-binding motifs, such as S_100 and EF-hand, are implicated in cellular regulatory mechanisms. UCH-L1’s peptidase_C12 motif is essential for ubiquitin-mediated protein turnover (Figure 5). These findings, presented in Tables 11 and 12, highlight the potential of these proteins as biomarkers in TBI pathophysiology and recovery processes.

**Table 11:** Motif analysis of GFAP, S-100B and UCH-L1 protein using MotifFinder and MotifScan servers

Proteins	Pfam_ID^a^	Description^b^	Position^c^	E-value^d^
GFAP	Filament	PF00038, Intermediate filament protein	376..68	3.1e−109
	Filament_head	PF04732, Intermediate filament head (DNA binding) region	66..7	2.1 e– 06
	DUF1664	PF07889, Protein of unknown function (DUF1664)	129.199 315..226	0.23 0.22
S-100B	S_100	PF01023, S-100/ICaBP type calcium binding domain	47..4	8.1 e– 22
	EF-hand_1	PF00036, EF-hand	80..54	3.7 e– 06
	EF-hand_7	PF13499, EF-hand domain pair	78..26	2 e– 05
	EF-hand_6	PF13405, EF-hand domain	79..59	0.01
	EF-hand_4	PF12763, Cytoskeletal-regulatory complex EF-hand	80..32	0.0035
	EF-hand_5	PF13202, EF-hand	78..55	0.011
	EF-hand_8	PF13833, EF-hand domain pair	79..43	0.045
	Spt20	PF12090, Spt20 family	63..25	0.076
UCHL1	Peptidase_C12	PF01088, Ubiquitin carboxyl-terminal hydrolase, family 1	204..5	1.1 e– 57

a Pfam ID: The identifier for the protein family in the Pfam database.

b Description: Briefly describe the pr otein family, including its function or characteristic features.

c Position: The range of amino acid positions in the protein associated with the respective Pfam ID.

d E-value: The statistical significance of the Pfam domain match; a lower value indicates a more significant match

**Table 12:** Post-translational modification site prediction of GFAP, S-100B, and UCH-L1 protein using PROSITE server

Proteins^a^	Category^b^	Signature^c^	Matching positions^d^
GFAP	RNA	IF_ROD_2	69–377
	Associated	*Intermediate filament (IF) rod domain profile*	
	Protein		
	Domain	IF_ROD_1	363–371		
	Posttranslational	*Intermediate filament (IF) rod domain signature*
	Modifications	
S-100B	RNA	EF_HAND_2	49 – 84
	Associated	*EF-hand calcium-binding domain profile*	
	Protein
	Domain	S100_CABP	57 – 78
	Posttranslational	*S-100/ICaBP type calcium-binding protein signature*	
	Modifications	EF_HAND_1	62 – 74
		*EF-hand calcium-binding domain*	
U-CHL1	RNA	UCH_1	84–100
	Associated	*Ubiquitin carboxyl-terminal hydrolase family one cysteine active-site*	
	Protein		
	Domain		
	Posttranslational		
	Modifications		

a Proteins: The name of the protein being analyzed.

b Category: The classification of the protein domain or its associated modifications, such as RNA-associated or posttranslational modifications.

c Signature: The specific domain signature associated with the protein, including the domain profile and its functional description.

d Matching positions: The protein’s range of amino acid positions corresponds to the identified signature

#### Post-translational modification site prediction using PROSITE server

Different signatures were identified across various locations within the proteins using the PROSITE database. In GFAP, these include the Intermediate Filament (IF) rod domain profile site spanning positions 69–377 and the IF rod domain signature site at 363–371 (Table 12). In S-100B, the EF-hand calcium-binding domain profile site was detected at positions 49–84, along with the S-100/ICaBP-type calcium-binding protein signature site at 57–78 and another EF-hand calcium-binding domain site at 62–74. For UCH-L1, the ubiquitin carboxyl-terminal hydrolase family 1 cysteine active-site was identified between positions 84–100 (Table 12).

### Identification, annotation, and analysis of domain architectures

The SMART server is an invaluable resource for exploring protein domain architecture and genetic modification. Our study, complemented by PredictProtein and SCOP data, analyzed the GFAP, S-100B, and UCH-L1 proteins, identifying disordered regions critical to their functionality.

GFAP is classified under SCOP’s superfamily of intermediate filament proteins, characterized by a coiledcoil region (Family: Intermediate filament protein, coiled-coil region). It shares a Fold known for its lefthanded parallel coiled-coil structure within the Class of all-alpha proteins. This classification includes proteins such as Prelamin-A/C, Vimentin, various Keratins, and Lamin-B.

S-100B belongs to the Family of S100 proteins, which adopt a Fold resembling a pair of EF-hands within the EFhand superfamily. This family includes S100-A4, S100-A8, S100-B, and other S100 variants, as well as Filaggrin.

UCH-L1 falls under the category of cysteine proteinases. The Superfamily of cysteine proteinases, with a Family specific to Ubiquitin carboxyl-terminal hydrolase UCH-L, includes two distinct Folds: the canonical cysteine proteinase catalytic core and a variant type. Notable proteins in this category include UCH-L1, UCH-L3, and YUH1 (Table 13).

**Table 13:** Structural classification of proteins using (SCOP SERVER)

Protein^a^	Superfamily^b^	Family^c^	Fold^d^	Class^e^
GFAP	Superfamily 3001560 — intermediate filament protein, coiled-coil region	Family 4003819 — intermediate filament protein, coiled-coil region	Fold 2000962 — Left-handed parallel coiled-coil	Class 1000000 – all alpha proteins
S-100B	Superfamily 3001983 — EF-hand	Family 4000919 — S100 proteins	Fold 2000120 – pair of EF-hands-like	Class 1000000 – all alpha proteins
UCH-L1	Superfamily 3001808 — cysteine proteinases	Family 4000880 — ubiquitin carboxyterminal hydrolase UCH-L	Fold 2001107 — a canonical type of cysteine proteinases catalytic core Fold 2001570 — variant types of cysteine proteinases catalytic core	Class 1000003 – alpha and beta proteins (a+b)

a Protein: The name of the protein being analyzed.

b Superfamily: The broader classification of the pr otein family based on structural and functional similarities.

c Family: The specific family within the superfamily, detailing the protein’s function.

d Fold: The s tructural classification of the protein, indicating the arrangement of its secondary structure elements.

e Class: The highest classification level is based on the p rotein structure

**Table 14:** Structural classification of GFAP, S-100B and UCH-L1 proteins using (SUPERFAMILY SERVER)

Protein^a^	Classification level^b^		Classification^c^	E-value^d^
GFAP	Superfamily	294–372	Intermediate filament protein, coiled-coil region	2.09 e– 23
		68–104	Intermediate filament protein, coiled-coil region	3.3 e– 11
		116–210	Myosin rod fragments	1.31 e– 2
	Family	294–372	Intermediate filament protein, coiled-coil region	3.43 e– 5
		68–104	Intermediate filament protein, coiled-coil region	6.6 e– 4
		116–210	Myosin rod fragments	0.012
S-100B	Superfamily	1–89	EF-hand	2.72 e– 24
	Family	1–89	S100 proteins	6.47 e– 5
UCH-L1	Superfamily	3–221	Cysteine proteinases	2.42 e– 76
	Family	3–221	Ubiquitin carboxyl-terminal hydrolase UCH-L	1.37 e– 9

a Protein: The name of the protein being analyzed.

b Classification level: The hierarchical level of structural classification, distinguishing between superfamily and family.

c Classification: The specific protein classification within its respective level indicates structural and functional similarities.

d E-value: The statistical significance of the classification, representing the likelihood of the match occurring by chance

The structural classification analysis of GFAP, S-100B, and UCH-L1 proteins revealed distinctive domain architectures and functional characteristics. For GFAP, multiple classifications were identified at both the superfamily and family levels. Within the superfamily classification, residues 294–372 were assigned to an intermediate filament protein with a coiled-coil region (*E*-value: 2.09e–23), while residues 68–104 exhibited a similar classification (*E*-value: 0.00000000000033). Another segment, spanning residues 116–210, was classified as myosin rod fragments (*E*-value: 0.0131). Consistent with these findings, the family classifications corroborated the intermediate filament protein classification for the same residue ranges, albeit with slightly different *E*-values.

For S-100B, a superfamily classification covering residues 1–89 identified an EF-hand motif (*E*-value: 2.72e–24), while the family classification recognized S100 proteins within the same range (*E*-value: 0.0000647). In the case of UCH-L1, a superfamily classification spanning residues 3–221 indicated cysteine proteinases (*E*-value: 2.42e–76), with the family classification assigning the protein as ubiquitin carboxyl-terminal hydrolase UCH-L within the same range (*E*-value: 0.00000000137) (Table 14).

This synthesis underscores the structural categorization and significance of these proteins within their respective superfamilies and families, highlighting their disordered regions that are critical for functionality. The study demonstrates the utility of domain architecture analysis in understanding protein function and potential genetic modification strategies. These insights, derived from the integration of SMART, PredictProtein, and SCOP data, provide a detailed understanding of the structural and functional aspects of these proteins, which are essential for future genetic research and manipulation. Structural classification tools such as CATH and SCOP were instrumental in establishing structure–function and evolution links to the GFAP protein and other proteins analyzed in this research. By analyzing domain architectures and understanding the roles of specific domains, researchers can gain valuable insights into the functions and mechanisms of these proteins, contributing to a broader understanding of traumatic brain injury biomarkers such as GFAP, S-100B, and UCH-L1.

### Pathway and systems biology analysis

**Figure 6:**
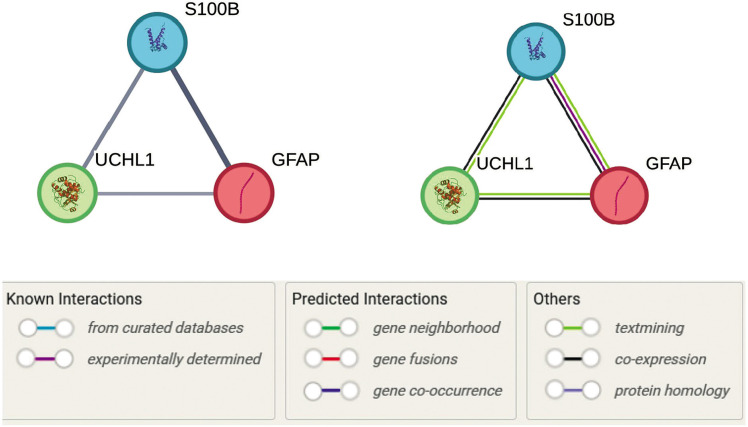
Protein–protein interaction network of GFAP, S-100B, and UCH-L1, generated using the STRING database, illustrates the functional relationships among these proteins in the context of traumatic brain injury (TBI). Nodes represent proteins, while edges represent interaction confidence scores, with thicker lines indicating higher confidence. GFAP and S-100B exhibit the strongest interaction (combined score: 0.925), supported by coexpression and experimental evidence, while S-100B and UCH-L1 show moderate interaction (combined score: 0.699). GFAP and UCH-L1 are linked with a lower confidence score (0.590), primarily supported by text mining. The network highlights the involvement of these proteins in TBI-related pathways, including neuroinflammation and ubiquitination. Pathway annotations are color-coded for clarity

The STRING analysis revealed a network of interactions among GFAP, S-100B, and UCH-L1, with direct connections supported by multiple lines of evidence (Figure 6). The interaction between GFAP and S-100B exhibited the highest confidence (combined score: 0.925), driven by co-expression patterns (score: 0.239) and experimentally validated interactions (score: 0.087). This pairing is biologically significant in the context of neuroinflammation, as both proteins are enriched in pathways such as Signaling by ERBB4 (Reactome: HSA-1236394) and Neuroinflammation (WikiPathways: WP5083), which play a crucial role in TBI-induced glial activation.

S-100B and UCH-L1 demonstrated a moderate interaction (combined score: 0.699), primarily supported by text mining (score: 0.671) and coexpression (score: 0.121). This interaction aligns with S-100B’s role in calcium signaling and UCH-L1’s function in ubiquitin-mediated proteolysis, as evidenced by its association with the Deubiquitination pathway (Reactome: HSA-5688426). The link between GFAP and UCH-L1, though weaker (combined score: 0.590), suggests a potential regulatory mechanism connecting GFAP’s structural role in astrocytes to UCH-L1’s protein degradation functions, particularly in pathways like Autophagy (Reactome: HSA-9612973) and Parkinson Disease (WikiPathways: WP2371), which are relevant to protein aggregation in TBI.

Pathway enrichment analysis highlighted the central role of neuroinflammation and ubiquitination in the network. GFAP and S-100B were strongly associated with immune response pathways, including Tolllike Receptor Cascades (Reactome: HSA-168898) and Glial Cell Differentiation (GO:0010001), while UCH-L1 was linked to protein homeostasis mechanisms such as the Ubiquitin-Proteasome System (KEGG: hsa05012).

## Discussion

This study establishes a foundational computational framework for analyzing GFAP, S-100B, and UCH-L1 as TBI biomarkers, leveraging state-of-the-art *in silico* tools to generate structural and functional insights. While the absence of experimental validation is acknowledged, the computational predictions align with recent advancements in structural biology, including AI-driven protein modeling, exemplified by the Nobel Prize-winning work on AlphaFold. This underscores the growing reliability of such methods in guiding biomedical research.

Advanced bioinformatics tools facilitate the structural analysis of GFAP, S-100B, and UCH-L1, revealing intricate details of their secondary structures and functional motifs. Structural bioinformatics analysis of GFAP identified a predominantly alpha-helical architecture (65%, 281 residues), complemented by minor beta-strands (6.2%, 25 residues) and coils (28.7%, 120 residues). This configuration underscores its role as a stable cytoskeletal protein essential for maintaining astrocytic integrity. Two conserved domains were identified: the Pfam00038 intermediate filament domain (residues 68–376; *E*-value: 1.12e–127) and the Pfam04732 filament head domain (residues 4–66; *E*-value: 2.51e–08). These domains, along with motifs such as Filament_head and DUF1664, highlight GFAP’s involvement in synaptic plasticity and axonal transport.

PTMs, including the IF rod domain (residues 69–377) and a bipartite nuclear localization signal (NLS), were computationally predicted, suggesting roles in DNA repair and nuclear shuttling during traumatic injury. Solvent accessibility analysis indicated that 63.89% of residues are buried, conferring proteolytic resistance and explaining GFAP’s persistence in biofluids postTBI.

Clinically, GFAP’s α-helix-rich structure aligns with its use in FDA-approved assays (e.g., BANYAN GFAP test) to reduce unnecessary neuroimaging in mild TBI cases. This structural stability (Gogishvili et al. 2024) enables reliable detection in serum and CSF. The study’s novel identification of the DUF1664 motif, previously uncharacterized in GFAP, opens avenues for investigating its role in neuroinflammation. AlphaFold-predicted models (TM-score: 0.92 ± 0.06; RMSD: 2.7 ± 2.0 Å) surpassed prior homology-based structures, offering atomic-level insights into GFAP’s interaction with inflammatory mediators such as IL-6 and TNF-α. These findings bridge structural predictions with experimental validations, including murine models demonstrating GFAP’s nuclear translocation during DNA damage (Posti et al. 2017).

S-100B exhibited a highly α-helical structure (67.39%, 63 residues) with no β-strands and 32.61% coils, consistent with its role as a calcium-sensing protein. The cd05027 S100B domain (residues 2–89; *E*-value: 1.68e–47) and EF-hand motifs (residues 49–84) were critical for Ca^2+^ binding and TLR4-mediated neuroinflammatory signaling. Buried residues (66.30%) stabilized Ca^2+^-binding pockets, while solvent-exposed regions mediated interactions with inflammatory receptors. Structural refinement using DeepRefiner yielded highaccuracy models (RMSD: 3.45 Å; TM-score: 0.99), resolving ambiguities in earlier SWISS-MODEL templates. A noncanonical coiled-coil region (residues 26–78), identified via ThreaDom analysis, suggests a scaffold for Ca^2+^-dependent oligomerization, a mechanism not previously described (Moreira et al. 2021; Michetti et al. 2023).

S-100B’s clinical relevance is underscored by its association with blood-brain barrier disruption, as demonstrated in multicenter studies (Mondello et al. 2021). Its EF-hand motifs align with experimental evidence showing S100B activation of TLR4/NF-κB pathways, exacerbating neuroinflammation in rodent models (Gupta et al. 2021). The study’s prediction of S100B’s coiledcoil domain provides a structural basis for its oligomerization, a feature implicated in amplifying inflammatory cascades. Furthermore, therapeutic targeting of S-100B using pentamidine, which reduces IL-6 and TNF-α *in vivo*, validates the computational insights into its Ca^2+^-binding pockets as druggable sites (Gupta et al. 2021).

UCH-L1 exhibited a balanced secondary structure, with 40.81% α-helices (91 residues), 17.94% β-strands (40 residues), and 41.26% coils (92 residues). The cd09616 Peptidase_C12 domain (residues 5–219; *E*-value: 3.16e–127) harbored a catalytic triad (Cys90, His161, Asp176) essential for ubiquitin hydrolysis. Solvent-exposed residues (42.60%) facilitated interactions with ubiquitinated proteins such as tau and α-synuclein, while buried regions stabilized the protease core. I-TASSER simulations revealed conformational flexibility in the ubiquitin-binding domain (residues 84–100), a feature undetected in static X-ray structures (PDB: 2ETL). Phylogenetic analysis positioned UCH-L1 within the cysteine protease superfamily, resolving its evolutionary divergence from UCHL-3 (bootstrap value: 98%) (Puri et al. 2024).

UCH-L1’s dual role in TBI – neuroprotection via aggregate clearance and neurotoxicity through excessive proteolysis – was corroborated by clinical studies. Its rapid release postinjury (detectable within 1 h) supports its inclusion in the ALERT-TBI diagnostic panel, which achieves 97% sensitivity for detecting intracranial lesions (Papa et al. 2016). The study’s dynamic modeling of UCH-L1’s catalytic triad provides mechanistic insights into its therapeutic modulation. For instance, inhibitors like 6-AMA have been shown to reduce axonal degeneration in vitro, aligning with computational predictions of UCH-L1’s role in tau aggregation (Yu et al. 2018). These findings highlight UCH-L1’s potential as a therapeutic target for mitigating secondary injury in TBI.

The focus on these three biomarkers was intentional, as they are well-characterized and clinically validated indicators of TBI, providing a targeted basis for future expansion to additional molecules. Though static models were employed, they offer critical preliminary insights into solvent accessibility, conserved regions, and PTMs, which can inform experimental designs for dynamic or microenvironment-specific studies. These computational findings serve as a roadmap for complementary experimental studies and clinical validation, ensuring a balanced integration of in silico and empirical approaches in advancing TBI biomarker research.

We recommend prioritizing experimental validation of the predicted structural and functional features to confirm their biological relevance. Future studies should expand the biomarker panel to include additional TBI-associated molecules, enhancing diagnostic specificity. Additionally, integrating multiomics approaches – including genomics, transcriptomics, and proteomics – could provide a holistic understanding of TBI mechanisms, effectively bridging computational predictions with clinical insights.

## Conclusions

**Figure 7:**
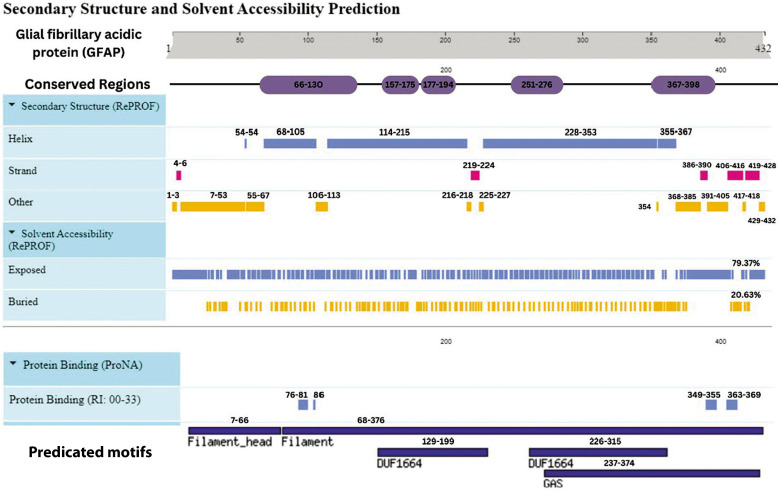
Integrative map of predicted results for GFAP. In line 1, the conserved regions are specified at 66–130, 157–175, 177–194, 251–276 and 367–398. Alpha Helix, Beta Strand, Other, Exposed and Buried from the secondary structure prediction servers, are specified in lines 2, 3, 4, 5, and 6. The result of protein binding site is specified in line 7. In the last, predicted motifs are specified in line 8

**Figure 8:**
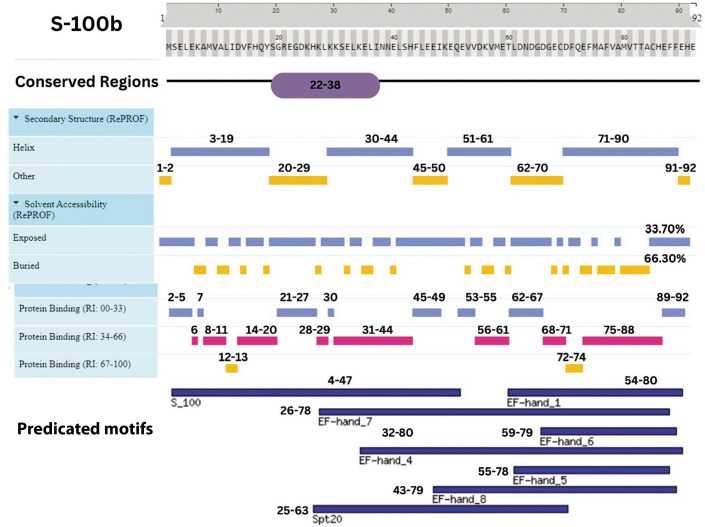
Integrative map of predicted results for S-100B. In line 1, the conserved regions are specified at (22–38). Alpha Helix, Beta Strand, Other, Exposed and Buried from the secondary structure prediction servers, is specified at line 2, 3, 4, 5, and 6. The result of protein binding site is specified in line 7. In the last, predicted motifs are specified in line 8

**Figure 9:**
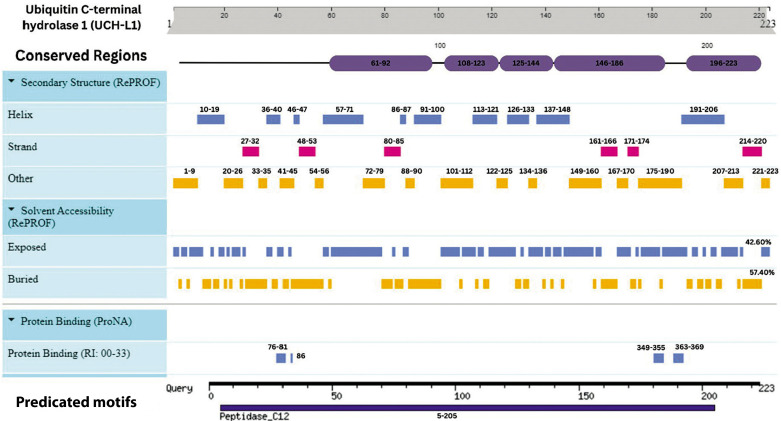
Integrative map of predicted results for UCH-L1. In line 1, the conserved regions are specified at (61–92, 108–123, 125–144, 146–186, and 196–223). Alpha Helix, Beta Strand, Other, Exposed and Buried from the secondary structure prediction servers, are specified in Lines 2, 3, 4, 5, and 6. The result of protein binding site is specified at line 7. In the last, predicted motifs are specified in line 8

This study leverages an integrated proteomic and bioinformatic framework to elucidate the structural and functional nuances of key TBI biomarkers: GFAP, S-100B, and UCH-L1. Through meticulous analysis, we have identified conserved regions, secondary structures, solvent accessibility, and PTM sites, enhancing our understanding of their structural models and domain architectures. Domain analysis positioned each protein within specific superfamilies, shedding light on domain-specific functions. The predominance of alpha-helices in GFAP and S-100B and a balanced mix of structural elements in UCH-L1 were confirmed, with solvent accessibility profiles indicating a majority of buried regions for GFAP and S-100B, whereas UCH-L1 displayed a more exposed structure (Figures 7–9).

Advanced bioinformatics servers facilitated the identification of protein binding motifs and structural features, with AlphaFold and I-TASSER providing the most accurate full-length tertiary structure predictions. Domain architecture analysis across various databases confirmed GFAP’s affiliation with the intermediate filament superfamily, S-100B’s with the EF-hand superfamily, and UCH-L1’s with the cysteine proteinase superfamily. These findings offer profound insights into the functional roles of these proteins in TBI pathophysiology.

The integrative approach adopted in this study not only deepens our comprehension of TBI biomarkers but also paves the way for the development of targeted diagnostic and therapeutic strategies, ultimately enhancing patient care.

Advanced bioinformatics servers facilitated the identification of protein binding motifs and structural features, with Alphafold and I-TASSER providing the most accurate full-length tertiary structure predictions. The domain architecture, analyzed through various databases, revealed GFAP’s affiliation with the intermediate filament superfamily, S-100B’s with the EF-hand superfamily, and UCH-L1’s with the cysteine proteinase superfamily. These findings offer profound insights into the proteins’ functional roles in TBI pathophysiology.

The integrative approach adopted in this study deepens our comprehension of TBI biomarkers and paves the way for the development of targeted diagnostic and therapeutic strategies, enhancing patient care.

## Supplementary material

Table S1: Proteins and its Functions: https://docs.google.com/document/d/1xQF-f-p7pGKB_m19haLsCnOuvWgI9rtKf7V_pK6tl4M/edit?usp=sharing

Table S2: Software used and its functionality: https://docs.google.com/document/d/17KUADcDOUwB18m0yXESD5kpU4_q4zFgg/edit?usp=sharing&ouid=109284365421778805632&rtpof=true&sd=true

Table S3: Evaluation and its scores: https://docs.google.com/document/d/17KUADcDOUwB18m0yXESD5kpU4_q4zFgg/edit?usp=sharing&ouid=109284365421778805632&rtpof=true&sd=true

Table S4: A summary of interaction scores and evidence types (e.g., experimental, text mining) extracted from string_interactions_short.tsv.https://drive.google.com/file/d/1B9m41rdJcc6G9_AMK96TpniIhwGf6qS-/view?usp=sharing.

Table S5: Pathway annotations from string_functional_annotations.tsv, detailing GO terms, Reactome, and WikiPathways entries associated with each protein. https://drive.google.com/file/d/1bNjTvLGu5MCPCYuMkn6BiDskQ37tBaPp/view?usp=sharing.
